# A Transformative Gold Patterning through Selective Laser Refining of Cyanide

**DOI:** 10.3390/nano11081921

**Published:** 2021-07-26

**Authors:** Jaemook Lim, Jimin Ham, Woohyun Lee, Eunseung Hwang, Won Chul Lee, Sukjoon Hong

**Affiliations:** BK21 FOUR ERICA-ACE Center, Department of Mechanical Engineering, Hanyang University, 55 Hanyangdaehak-ro, Sangnok-gu, Ansan 15588, Korea; limjaemook@hanyang.ac.kr (J.L.); jiminham@hanyang.ac.kr (J.H.); woohaha94@hanyang.ac.kr (W.L.); joseph5017@hanyang.ac.kr (E.H.)

**Keywords:** selective laser refining, gold cyanide, selective laser sintering, gold electrode, electrode repair

## Abstract

Gold is an essential noble metal for electronics, and its application area is increasing continuously through the introduction of gold nanoparticle ink that enables rapid prototyping and direct writing of gold electrodes on versatile substrates at a low temperature. However, the synthesis of gold nanoparticles has certain limitations involving high cost, long synthesis time, large waste of material, and frequent use of chemicals. In this study, we suggest simultaneous laser refining of gold cyanide and selective fabrication of gold electrodes directly on the substrate without a separate synthesis step. Gold cyanide is commonly the first product of gold from the primitive ore, and the gold can be extracted directly from the rapid photothermal decomposition of gold cyanide by the laser. It was confirmed that laser-induced thermocapillary force plays an important role in creating the continuous gold patterns by aligning the refined gold. The resultant gold electrodes exhibited a low resistivity analogous to the conventional direct writing method using nanoparticles, and the facile repair process of a damaged electrode was demonstrated as the proof-of-concept. The proposed transformative approach for gold patterning, distinguished from the previous top-down and bottom-up approaches, has the potential to replace the well-known techniques and provide a new branch of electrode manufacturing scheme.

## 1. Introduction

Gold is widely used as a form of micropatterned layers for electrical, optical, and biosensing applications. In the formation of Au micropatterns, many people focus only on microfabrication techniques, but Au refinement is also an important starting point of the micropattern formation. In the refinement process (also known as the cyanide process), Au is extracted from low-grade ore by converting the gold to a water-soluble complex, requiring multiple steps and high-tech equipment accompanied with some environmental alerts. The drawbacks found in its refinement process, for example, process complexity and costly equipment, even escalate for the fabrication of Au micropatterns. In general, the Au film is formed by sputtering [1], evaporation [2], and chemical vapor deposition [3,4], and then the wet etching [5], photolithography [6,7], and lift-off [8] techniques are usually conducted for patterning of the Au electrode on the pre-fabricated Au film, which are well-known to be time-consuming and high-cost procedures.

The above subtractive fabrication methods have intrinsic limitations regarding the inevitable waste of noble materials and process complexity, especially in the case that the actual regions of interest are smaller than the entire target substrates, e.g., conductor repair for damaged or missing circuits. To enhance the efficiency of the fabrication method, direct writing techniques that create Au patterns directly only on the target region are proposed [9]. Subsequently, with the advances of nanoparticle (NP) synthesis techniques, direct writing [10,11] and rapid prototyping [12] have drawn keen attention. In this context, selective laser sintering [11,13] (SLS) and laser-induced transfer [14] have received a large amount of attention thanks to their diverse advantages allowing for frequent change of the pattern design and rapid fabrication without additional cleaning [15]. However, same as for the conventional patterning techniques, such as wet etching and photolithography, the direct writing techniques based on Au NPs also require separate synthesis steps which result in not only an increase in cost and time but also plenty of material waste due to the low yield. Moreover, a large amount of chemical use for the synthesis urges the development of eco-friendly techniques [16].

**Figure 1:**
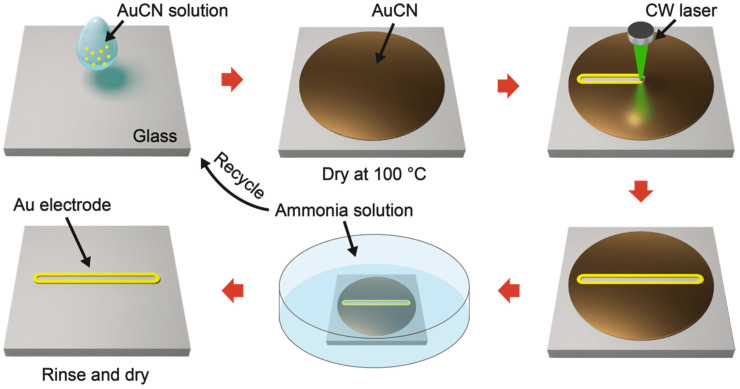
Schematic drawing of a transformative gold patterning through selective laser refining of cyanide: AuCN dissolved in ammonia solution is drop-casted and dried on the glass substrate. After the laser processing, the residual AuCN is rinsed, and the Au electrode is selectively fabricated as a resultant.

Following these facile and on-demand fabrication trends, we devised transformative gold patterning through selective laser refining of gold cyanide (AuCN). The AuCN, the first product of the cyanidation process, can be easily transformed into Au as well as desolved in aqueous solutions of strong bases [17,18,19,20,21,22]. Based on these characteristics, we provide a facile fabrication method for Au micropatterns directly from AuCN without separate synthesis steps, derived from the laser-induced in situ refinement and alignment (Figure 1). The proposed process is expected to reduce the cost and time of additional refinements and also possesses the potential for encouraging further transformative fabrication techniques that convert the raw material into the final target materials directly at the substrates [23,24].

## 2. Materials and Methods

### 2.1. Sample Preparation

The conventional drop-cast method was utilized to deposit gold cyanide (AuCN, purchased from Strem Inc., Newburyport, MA, USA) on the surface of the target substrate. In this experimental setup, the glass (microscope slides of 1 mm thickness, Marienfeld, Lauda–Königshofen, Baden–Württemberg, Germany) was selected. An aqueous solution of 150 mM AuCN (dissolved in an ammonia solution, no more dissolved solute dispersed in solution) was dropped on the glass substrate and dried on a hot plate for 10 min at 100 °C. After the fabrication of the Au electrode, the remaining AuCN was wet etched with a 14.8 M ammonia solution for 15 min. The Au electrode was cleaned using DI water several times, then dried for 10 min at 100 °C.

### 2.2. Optical Setting

The optical setup incorporated a 532 nm wavelength CW diode-pumped solid-state (DPSS) laser Lighthouse Photonics Sprout-G-5W, which uses Nd:YVO4 as its gain medium. Its spatial mode (Gaussian mode) was TEM00 at M2 = 1.0–1.1, with a beam diameter of 2.3 mm ± 10%. In the current study, the laser was focused with a Mitutoyo M Plan Apo 5× (Kawasaki, Japan) objective lens, and the laser scanning process was conducted using an Aerotech ANT130-060-XY-25DU-XY-CMS-MP-PLUS motorized 2-axis translational stage (PA, USA) by programming the scanning paths. The laser power was controlled between 0.10 and 2.00 W at scanning speeds of 150–1350 μm/s to assess the effects of the laser conditions on the selective laser refining of AuCN with thermocapillary force.

### 2.3. Measurement (SEM, AFM, and Resistivity)

Optical microscope (BX53M, Olympus, Tokyo, Japan) and field emission scanning electron microscopy (FE-SEM) (MIRA3, TESCAN) were used for capturing the microscope images of the Au electrodes. The electrical resistance and the cross-sectional profiles of the Au electrode were obtained using a multimeter (TK-3205, Taekwang Co., Busan, Korea) and atomic force microscopy (AFM) (XE0-100, Park Systems, Brno, Czech Republic), respectively. The electrical conductivity of the fabricated Au electrode was calculated using the measured electrical resistances and cross-sectional profiles.

### 2.4. Laser-Induced Temperature Field Simulation

For the qualitative analysis of effects derived from the laser spot size and laser power, the finite element method (FEM) model was simplified as a 100 μm thin film on the 1 mm glass substrate. For the thermal conductivity of a thin film, 100 W/m·K was used. The result of the FEM analysis only indicates the change of the temperature gradient according to the laser spot size is more significant than according to the laser power. The representative laser power was set as 1.5 W. The scanning speed was fixed at 150 μm/s. The diameters of a deposited laser beam (TEM_00_ Gaussian distribution) were 20 μm, 100 μm, 200 μm, and 1000 μm. The temperature data were collected from the central elements perpendicular to the scanning direction.

### 2.5. Application (Repair and Adhesion Test)

The damaged metal substrates for the repair test were prepared by artificially cracking a Pt-coated glass substrate and an Au-coated glass substrate with a sharp cutter. The platinum and gold on the glass wafer (purchased from the Find Chemical Industry) were coated at a thickness of 30 nm and 100 nm (with an additional 10 nm layer of chromium below) using an e-beam evaporator (FE-EB10, ULVAC, Inc., Methuen, MA, USA). The adhesion test was conducted under the repeated peeling in the same place on the surface of the electrode with adhesive tape (3M, Scotch).

## 3. Results and Discussion

In this study, we introduce an Au electrode fabrication technique that is based on the selective transformation of primitive material at the target spot to drastically reduce the time and cost spent on the synthesis of NPs for the fabrication of electrodes. This facile fabrication method follows the procedures illustrated in Figure 1. We selected AuCN as a target material for the fabrication of Au electrodes through simultaneous refinement and thermocapillary-induced alignment at the target region. AuCN, an inorganic material that contains linear Au–C–N chains [25], is the early product from the extracted ore, and it can be considered as a raw material of refined Au products. AuCN can be decomposed to form metallic gold by various energy sources including heat, electron beams, and ion beams [18,21,22]. Therefore, we can induce the fabrication of Au structures on the target substrates through the minimal coating and selective laser-induced photothermal heating procedures of AuCN.

The proposed technique possesses the characteristics of SLS, one of the promising rapid-prototyping techniques. The laser is used as a heat source in these techniques, enabling the precise control of spatial and temporal temperature gradients at the target [26]. The photothermal reaction by the continuous wave (CW) laser thermally decomposes AuCN and also partially melts the surface of created Au layers. The locally sintered Au is accumulated at the edge of the laser scanning path due to the thermocapillary force. After the fabrication of the Au electrode, the non-irradiated region of the AuCN layer remains on the substrate, but these residuals can be easily wet-etched using ammonia solution. Due to the good solubility of AuCN in the ammonia solution [18,19,20] and the volatility of the ammonia solution, this AuCN solution can be recycled after the etching procedure as the drop-casting solution to minimize the material waste [27].

With the successful selective laser refining of AuCN, we can fabricate the Au pattern directly on the glass substrate. The proposed technique presents significant differences from the conventional SLS of Au NPs. The main reason is attributed to direct uses of raw material that contains unnecessary substances as a final product. In the conventional SLS process using Au NPs, the solvent, as well as the capping agent of Au NP ink, are easily evaporated. At the same time, the surfaces of dense Au NPs are partially melted and agglomerates into a continuous conductive path. However, in the case of AuCN, which is distinguished from the Au NP ink, the molar volume of a single Au atom is only 32.677 percent of the molar volume of a single AuCN molecule, while the rest of the volume is occupied with cyanide ligand (i.e., C and N). This characteristic of raw material results in a different outcome once the conventional SLS is applied to the AuCN thin film. The thermal decomposition of cyanide by laser irradiation creates numerous voids and disconnects the network of the Au which yields discrete Au nanoislands instead of a continuous conductive path. Thus, to overcome this limitation and obtain the electrically conductive layer, an additional agglomeration of refined Au is necessary. We have devised that extreme temperature gradient by intensive laser irradiation assists in aligning and gathering the refined Au nanoislands [28].

**Figure 2:**
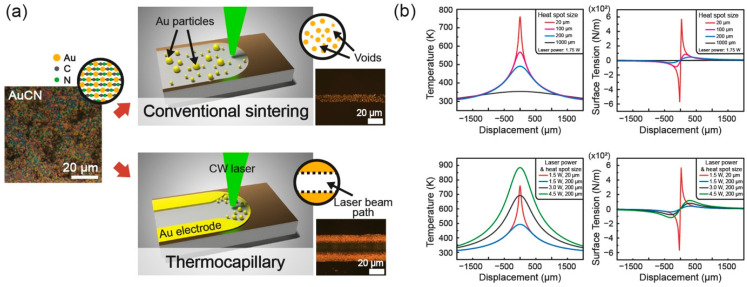
(**a**) Illustrations of the difference between the proposed thermocapillary-assisted selective laser refining and SLS and (**b**) the FEM analysis of the temperature gradient dependent on the laser spot size and the laser power as well as the calculated surface tension representing the resultant thermocapillary force (upper graphs: the effect from different laser spot size; lower graphs: the effect from different laser power).

The thermocapillary force induced by the intensive photothermal reaction is often used as post-processing to eliminate the defects of the product from rapid prototyping techniques [29,30]. We also expected that the intrinsically porous Au patterns coud be gathered and re-agglomerated by the high spatial gradient of temperature as schematically shown in Figure 2a. During the conversion of AuCN to Au, the slightly melted Au migrated to both sides of a laser beam path by the laser-induced thermocapillary force as confirmed in the inset optical image. To analyze the effects of the laser spot size and the laser power (in FEM analysis, the scanning speed was fixed at 150 μm/s), FEM analysis was conducted. As analyzed in the upper graphs of Figure 2b, the laser spot size had a great influence on the thermocapillary force. When the heat became localized, the thermocapillary force was enhanced exponentially and concentrated at the center of the heat spot, which acted in a laterally outward directions (i.e., fsollowing the surface tension direction, away from the center). On the contrary, the effect from the increase in laser power to the resultant thermocapillary force was not as significant: the thermocapillary force improved when the laser power increased (lower graphs, Figure 2b), but their effect was rather distributed over a wide range. This result of the FEM simulation was further developed with the following formula [31]:

(1) $$\tilde{\tau }\cdot \hat{n}=\nabla \sigma =\frac{\partial \sigma }{\partial T}\nabla T$$

where $\tilde{\tau }$, $\hat{n}$, σ, and T were sequentially shear stress, surface normal, surface tension, and temperature. As stated in this formula, the thermocapillary force was dependent on the temperature gradient. In this sense, the spot size subject to the temperature rise is the critical parameter to induce the thermocapillary flow, and laser processing has a superior characteristic due to the fact of its localized and rapid photothermal reaction. The selective laser refining process of AuCN and the subsequent agglomeration utilizing laser-induced thermocapillary force are naturally combined for the in situ transformative fabrication of Au electrodes. Hence, the characteristics of laser processing are essential to enable the proposed technique to create a continuous conductive path.

**Figure 3:**
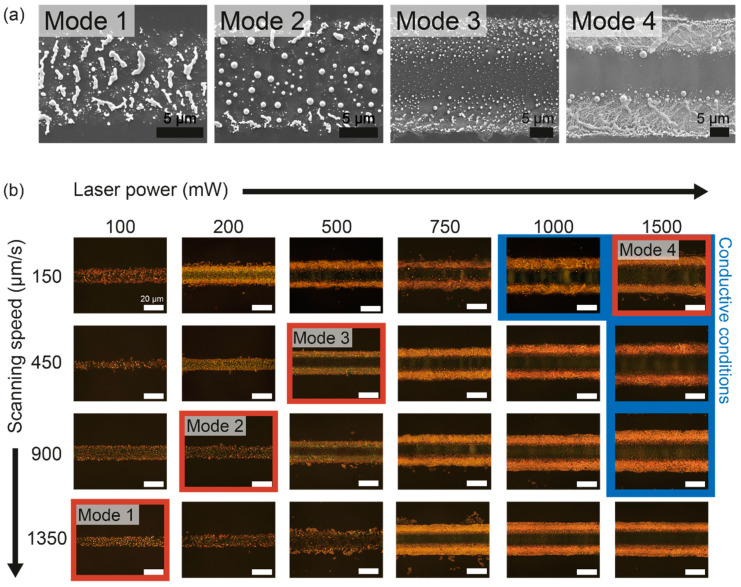
Evolution of the Au pattern depending on the laser intensity: (**a**) representative modes of the results of laser selective refining (SEM images); (**b**) optical images of fabricated Au patterns by the selective laser refining according to laser intensity and resulting thermocapillary force.

As shown in Figure 3a, the results of the proposed method are largely classified into four modes depending on the laser conditions. The SEM images of each mode describe their characteristics. In the case of mode 1, AuCN is thermally decomposed at its originally coated region by laser irradiation similar to the conventional SLS of Au NPs. However, due to the complexity of the chemical composition of the target material, the resultant Au patterns are disconnected from each other by the vaporization of cyanide that belongs to a large portion of the original material, and the resultant is inappropriate to perform as an electrode. Such occurrence of the voids was even enhanced in mode 2, as the decomposed Au were accumulated on Au islands at the center due to the higher laser intensity than mode 1. As the laser intensity increased further, the thermocapillary force in conjunction with the prolonged melting time started to affect the morphology of the decomposed Au as illustrated in mode 3. The Au particles in the center of the lateral direction were heated more intensively and, at the same time, the thermocapillary force pushed these Au particles to the borders of the laser beam path. However, despite the occurrence of thermocapillary flow, some Au was still widely distributed from the center to the edges in the form of particles, and a significant portion between the Au particles was composed of voids, which is insufficient for Au electrodes overall. Once the thermocapillary force is further escalated, the Au patterns transform into the form of the continuous conductive path as shown in mode 4, although including the empty space in the center of the lateral direction. The sufficient temperature gradient obtained at the adequate laser spot size and laser intensity shapes continuous and conductive double Au electrodes to both sides of the laser scanning path.

With the fixed laser spot size, the changes in the Au’s morphology according to the laser intensity are further illustrated in Figure 3b. Below the laser intensity of mode 1 (laser power: 150 mW, scanning speed: 1350 μm/s), the AuCN failed to maintain the continuum, while the thermally undecomposed AuCN remained on the laser path (Figure S2). The surface melting and the subsequent sphericalization of the particles at the central region were more prominent as the laser power increased or the scanning speed decreased (i.e., maximum temperature increase), which was apparent in mode 2 (laser power: 200 mW, scanning speed: 900 μm/s). When the laser intensity was above mode 2, the thermocapillary force was reinforced gradually, and the yellow Au lines were created at both lateral sides of the Au pattern. Consequently, in mode 3 (laser power: 500 mW, scanning speed: 450 μm/s), the Au particles were spread out to both sides and the lateral center appeared relatively empty. Nonetheless, in contrast to the optical images showing the two distinct boundaries near the lateral center, the SEM images in Figure 3a demonstrate that the resultant Au still consisted of particles instead of a continuous film. As the laser intensity approached mode 4 (laser power: 1500 mW, scanning speed: 150 μm/s), the boundaries at both sides of the Au lines became more clear due to the formation of the Au network. These Au networks enabled the electrical conductivity of the resultant Au electrodes (inset: conductive conditions in Figure 3b). We expect that the microscopic roughness of the Au electrode might find its usage as energy applications that prefer a large surface area such as a supercapacitor [32,33,34]. At a laser power >2000 mW, the excessive temperature and its gradient usually lead to a thermal failure of the glass substrate (Figure S1), while the effect of the thermocapillary force is already sufficient to create Au electrodes.

**Figure 4:**
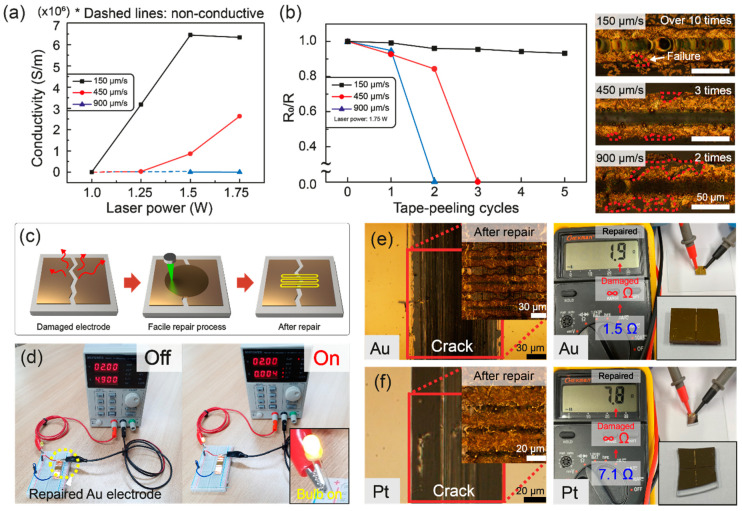
Characterizations of the fabricated Au electrodes by selective laser refining: (**a**) electrical conductivity of the Au electrodes according to the laser condition; (**b**) adhesive tape tests of Au electrodes according to the different scanning speeds of 150 μm/s, 450 μm/s, and 900 μm/s at 1.75 W and measurements of relative resistances R_0_/R (R_0_: the initial resistance, R: the resistance after tape–peeling cycle) against repeated tape–peeling cycles (inset: optical images of Au electrode after iterative adhesive tape tests), and demonstrations and measurements of the facile repair method to damaged electrodes; (**c**) Schematic images of facile repair process using selective laser refining; (**d**) photographs of the circuit using a repaired Au electrode by the proposed method, optical images of both completely disconnected and then repaired (**e**) Au and (**f**) Pt electrodes, and the resistance measurements of the repaired electrode, respectively (original resistance of Pt: 7.1 Ω and Au: 1.5 Ω, repaired resistance of Pt: 7.8 Ω and Au: 1.9 Ω).

The electrical properties of the resultant Au electrodes in terms of electrical conductivity were calculated using electrical resistances and cross-sectional areas according to the laser power at different scanning speeds as shown in Figure 4a. The electrical conductivity started to appear at sufficient laser intensity, and it increased at the lower scanning speed owing to the increase in the laser fluence that induced more complete refinement of the as-prepared AuCN into conductive Au. However, although the thermocapillary force increased further with the laser intensity, the electrical conductivity of the final Au electrode became saturated, as shown in the conditions of 150 μm/s at 1.5 W and 1.75 W, since the laser processed patterns possessed only a limited portion of Au, and the Au electrode had already been formed by sufficient laser intensity. The electrical resistivity of the fabricated Au electrode was measured to be 1.551 × 10^−7^ Ω·m at the 150 μm/s scanning speed with 1.5 W laser power, which is approximately 6.4 times higher than the resistivity of bulk gold (2.44 × 10^−8^ Ω·m). For further investigation of the fabricated Au electrode as the circuit restoration purpose, the adhesive tape test was conducted at the electrically conductive conditions of 1.75 W. The resistance (R) measured after pulling off the tape gradually decreased compared to the initial resistance (R_0_), according to the tape–peeling cycles. The Au electrodes produced at the scanning speed of 900 μm/s and 450 μm/s endured only a few adhesive tape tests, showing easy delamination of some portions from the resultant Au electrode as shown in the inset of Figure 4b. Whereas for the 150 μm/s condition, the Au electrode demonstrated its enhanced robustness against the adhesive tape test as shown in Figure 4b. The relative resistances (R_0_/R) according to the number of adhesion tests were 99.2%, 95.9%, 95.6%, 94.2%, and 93.3% of the initial resistance, respectively. After 10 performances of the identical adhesive tape test, the Au electrode lost its electrical conductivity. This result is contrary to the noble metal electrodes fabricated by vacuum evaporation techniques, such as gold, silver, and platinum, that require an additional adhesion layer such as chromium [35] on the glass substrate to improve the adhesion between the glass and noble metal. The durability of the Au electrodes was augmented as the laser intensity increased, and we expected that the thermal modification of the underlying glass substrate would escalate the roughness of the glass surface and generate the mechanical interlocking [36] between the two surfaces. As the laser power reached 2 W at 450 μm/s, the hollow line of the thermally damaged glass occurred on the glass substrate by the excessive thermal effect (Figure S1), which led to the inference that the precise control of the thermal effect at the glass surface can be used for the facile fabrication of noble metal directly on the glass substrate through simultaneous selective laser refining in the future.

For the practical application of selective laser refining of AuCN, the facile repair of the damaged electrode is demonstrated and characterized in Figure 4c–f. The proposed method has the following advantages: (1) The AuCN solution, which is prepared without complex synthesis steps, is selectively drop-casted at the target area, and it is expected that the AuCN solute etched after the laser process can be recycled with the control of concentration. (2) Being a solution process using a visible CW laser source, the entire process can be monitored to obtain the required electrical resistance. (3) The overall restoration process and the required equipment are much simpler as a result. A future portable repair kit can be configured following the provided concepts (Figure 4c). On the damaged and the consecutively non-conductive region of the electrode, the ammonia solution of AuCN is selectively drop-casted and dried, followed by the selective laser refining process. The number of laser scanning is controlled to match the resistance of the repaired electrode to be analogous to the original resistance. The photographs in Figure 4d show that the Au electrode repaired using selective laser refining of AuCN was restored as a conductor. The electrical resistances of the repaired Au and Pt electrodes are measured in Figure 4e,f, which have analogous electrical resistances to the original electrodes.

## 4. Conclusions

The proposed Au patterning technique using selective laser refining of AuCN suggests the direct refinement of raw material and facile fabrication of electrodes, directly on the target substrates. This transformative approach, which is distinguished from the bottom-up approach via conventional gold nanoparticles and also the top-down approach derived from subtractive fabrication techniques including photolithography, provides a new fabrication scheme for Au electrodes. Furthermore, this in situ simultaneous refinement and alignment technique is also expected to synergize with recent trends in rapid prototyping techniques requiring facile fabrication and frequent changes in design and evolution into field-tailored technologies. As demonstrated in the concept of in situ repairment of the damaged electrode, the resultant of the current study can provide a comparable electrical conductivity to the original substrate; thus, we predict the selective laser refining technique will be effective for other raw materials that embrace metal elements.

## Supplementary Materials

The following are available online at https://www.mdpi.com/article/10.3390/nano11081921/s1, Figure S1: Optical image of the thermally damaged glass substrate at the laser condition of 2 W and 450 μm/s., Figure S2: Comparison of before and after etching process using ammonia solution; (a,b) Optical images of Au electrodes on mode 1 and 4, respectively. (c) SEM images of Au electrodes on mode 4.
