# Supplementary material for: A Transformative Gold Patterning through Selective Laser Refining of Cyanide
Source: Nanomaterials (Basel). 2021 Jul 26;11(8):1921. doi: 10.3390/nano11081921 (PMC8400824; doi:10.3390/nano11081921)
Supplement: Supplementary file 1 [file nanomaterials-11-01921-s001.zip › nanomaterials-1288812-supplementary.pdf]

Supporting Information

A Transformative Gold Patterning through Selective Laser Refining of Cyanide

Jaemook Lim †, Jimin Ham †, Woohyun Lee, Eunseung Hwang, Won Chul Lee *, and Sukjoon Hong *

Department of Mechanical Engineering, BK21 FOUR ERICA-ACE Center, Hanyang University,
55 Hanyangdaehak-ro, Sangnok-gu, Ansan, Gyeonggi-do 15588, Korea;
limjaemook@hanyang.ac.kr (J.L.); jiminham@hanyang.ac.kr (J.H.);
woohaha94@hanyang.ac.kr (W.L.); joseph5017@hanyang.ac.kr (E.H.)

* Correspondence: wonchullee@hanyang.ac.kr (W.C.L.); sukjoonhong@hanyang.ac.kr (S.H.); Tel.: +82-31-400-5257 (W.C.L.); +82-31-400-5249 (S.H.)

† These authors contributed equally to this work.

Supplementary Note 1: The main field equations and boundary conditions for the FEM analysis

The main field equations are general heat conduction equation and Gaussian power distribution, built-in settings of 'Heat Transfer Module: Deposited Beam Power' mode, expressed as

$$\frac{\partial}{\partial x}\left(k\frac{\partial T}{\partial x}\right)+\frac{\partial}{\partial y}\left(k\frac{\partial T}{\partial y}\right)+\frac{\partial}{\partial z}\left(k\frac{\partial T}{\partial z}\right)+\dot{e}_{gen}=0 \qquad (1)$$

where k and T implies conduction coefficient and temperature while the energy generation ($\dot{e}_{gen}$) is given by the laser power input, which is distributed as

$$f(\boldsymbol{o},\boldsymbol{e})=\frac{1}{2\pi\sigma^2}exp\left(-\frac{d^2}{2\sigma^2}\right),\ d=\frac{\|\mathbf{e}\times(\boldsymbol{x}-\mathbf{o})\|}{\|\mathbf{e}\|} \qquad (2)$$

where o, e, x, and σ are beam origin point vector, beam orientation vector, spatial coordination vector and standard deviation of the laser beam. In the steady scanning condition, the constant d generally depends on the scanning speed (v) and time (t), defined as

$$d=vt \qquad (3)$$

since the components are set as $\mathbf{o}=[vt,0,0]$, $\mathbf{e}=[0,0,-1]$, and $\boldsymbol{x}=[0,0,0]$.

For the boundary conditions, an ambient temperature (293.15 K) is applied at the bottom and the natural convection coefficient (h = 5 m^2 K) is applied on the other boundaries.

The temperature profile is calculated using FEM simulation, but the surface tension is obtained from Equation (1), by multiplying the temperature coefficient of the surface tension (∂σ/∂T; -0.1773 × 10-3 N/m·K of liquid gold) with the temperature gradient obtained from the FEM simulation.

Supplementary Note 2: Resultant Au structures on the glass substrate depending on laser conditions.

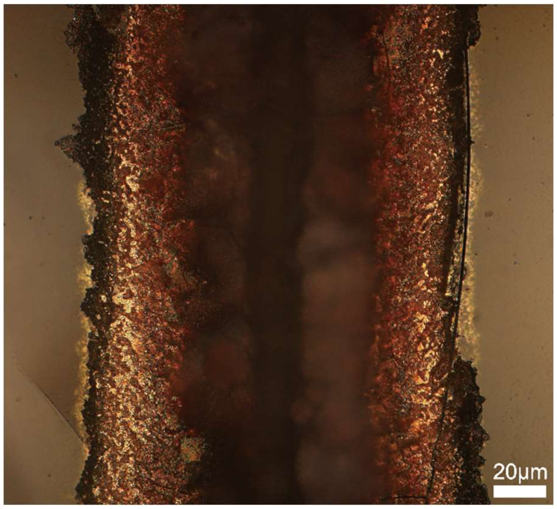

Figure S1. Optical image of the thermally damaged glass substrate at the laser condition of 2 W and 450 μm/s.

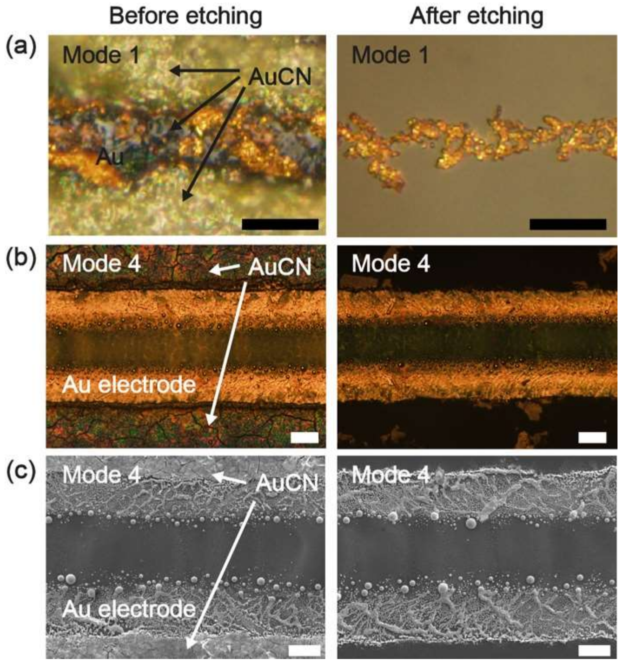

Figure S2. Comparison of before and after etching process using ammonia solution; (**a**,**b**) Optical images of Au electrodes on mode 1 and 4, respectively. (**c**) SEM images of Au electrodes on mode 4.
